# BLITSFR: scalable comparative genomics with self-contained interactive single file report

**DOI:** 10.1093/bioadv/vbag282

**Published:** 2026-09-25

**Authors:** Budi Permana, Thom Cuddihy, Brian M Forde

**Affiliations:** Institute for Molecular Bioscience, The University of Queensland, Brisbane, Australia; Australian Infectious Diseases Research Centre, The University of Queensland, Brisbane, Australia; Institute for Molecular Bioscience, The University of Queensland, Brisbane, Australia; Australian Infectious Diseases Research Centre, The University of Queensland, Brisbane, Australia; Institute for Molecular Bioscience, The University of Queensland, Brisbane, Australia; Australian Infectious Diseases Research Centre, The University of Queensland, Brisbane, Australia

## Abstract

**Motivation:**

Comparative genomics routinely requires the alignment of query genomes against a reference to delineate conserved and variable regions, yet existing tools often produce static visualisations or necessitate centralised web services and data uploads.

**Results:**

To address this, we present BLITSFR (BLAST Interactive Tracks in Single-File Report), a command-line tool that compares query genomes against a reference and returns a single interactive HTML file. The report opens in any modern browser, requiring no server or data upload, and lets users explore conserved and variable regions across the genomes. BLITSFR handles both assembled genomes and raw reads, and processes hundreds of genomes in minutes on a standard laptop with genome tracks sorting, filtering, and colouring by metadata are performed within the report, without requirement for re-running the analysis. We demonstrate its utility by analysing vancomycin-resistant *Enterococcus faecium* ST78 genomes to confirm the conservation of Tn1549-like transposons and a case of β-lactam-resistant *Corynebacterium diphtheriae*, where coverage depth resolved resistance transposon copy number.

**Availability and implementation:**

BLITSFR and SCIFR are freely available at the provided GitHub repositories (https://github.com/nalarbp/blitsfr, https://github.com/nalarbp/scifr).

## 1 Introduction

Increasing volumes and complexity of genomic data present significant challenges in interpreting results and communicating findings (Tao *et al.* 2004, O’Donoghue 2021). In comparative genomics, query genomes are commonly compared against a reference to delineate conserved and variable regions that distinguish one strain from another. These comparisons are conventionally displayed as genome maps, often in circular or linear formats, where variation in the query genome is displayed as presence, absence, or depth coverage relative to the reference (Stothard *et al.* 2019). At the scale of hundreds of genomes, static maps often limit user experience in exploration and finding interesting regions, as track order, colouring, and filtering are often fixed at the point of rendering. Therefore, bioinformatics tools increasingly feature interactive reports to enhance data exploration and interpretation. For example, MultiQC (Ewels *et al.* 2016), Krona (Ondov *et al.* 2011) and Clinker (Gilchrist and Chooi 2021) compile results from analyses into interactive files, enabling users to efficiently review and interrogate diverse datasets.

The CGView viewers has been widely used for generating and visualising genome comparison maps, underpinning tools such as BRIG (Alikhan *et al.* 2011), CCT (Stothard *et al.* 2019), CGView Server (Grant and Stothard 2008), and Proksee (Grant *et al.* 2023). BRIG (Alikhan *et al.* 2011) renders CGView output through a graphical desktop interface, in which the reference, query set, and display parameters must be specified before each run. CCT (Stothard *et al.* 2019) uses the same Java renderer but with a command-line interface to streamline thousands of comparison genomes. However, both generate static images and require time-consuming manual configuration prior to analysis. Proksee (Grant *et al.* 2023) introduced CGView.js (Grant and Stothard 2026), a JavaScript reimplementation of CGView that supports interactive maps and zooming to base resolution. However, Proksee operates as a centralised web service requiring data upload to remote servers, with limited batch processing for large number of genomes, which precludes analysis on local resources.

Common strategies for bioinformatics interactive reporting typically fall into two categories: static report generation and dynamic web applications. Static approaches, exemplified by MultiQC (Ewels *et al.* 2016), and Clinker (Gilchrist and Chooi 2021), use template engines such as Jinja to process data through predefined templates and generate HTML files. Web applications, such as Proksee and Shiny-based applications (Argimon *et al.* 2016, Argimon *et al.* 2021, Grant *et al.* 2023, Chang *et al.* 2024), offer greater interactivity but require a web server, which often complicates integration with typical HPC-based bioinformatics pipelines. Recent advances in single-page application (SPA) frameworks bridge these two approaches: by handling routing and view updates client-side, an entire application can be bundled with its data into a single HTML file that runs in the browser without server infrastructure (Hadfield *et al.* 2018, Permana *et al.* 2024). This client-side execution reduces latency, optimises resource utilisation, and simplifies deployment, as the application can be hosted on static file servers or shared as standalone reports.

Here, we present BLITSFR (BLAST Interactive Tracks in Single‑File Report), a Nextflow-based command-line tool designed for scalable comparative genomics that characterises genomic regions variation through parallel processing and visualises it via a standalone, interactive single-file report. BLITSFR is built on SCIFR, a novel design pattern that leverages Single Page Application (SPA) architecture to generate self-contained, interactive reports capable of complex client-side state management, delivering web-application functionality without a web server. By combining Nextflow’s parallel processing with SCIFR’s client-side interactivity, BLITSFR efficiently handles large-scale genome comparisons while producing a rich exploration platform for local execution and resource utilisation, and providing a reusable framework for bioinformatic application development.

## 2 Methods

### 2.1 Development of self-contained interactive single-file reporting system

BLITSFR is built on a reusable novel design pattern that exploits SPA architecture as a templating system, referred to as Self-Contained Interactive File Report (SCIFR). The SCIFR system comprises three core components: the *Viewer*, the *Processor*, and the *Mutator* (Fig. 1a). The SPA template functions as the *Viewer*, responsible for rendering outputs generated by the *Processor*. The *Processor* can be a simple script or a complex pipeline that generates output for presentation in the *Viewer*. The *Mutator* serves as a bridge between the *Processor* and the *Viewer* by identifying input placeholders within the template and replacing them with the *Processor’s* output, thereby producing the final HTML report. This approach is inspired by plasmid cloning, where a data fragment (analogous to a gene of interest) can be dynamically inserted into the template backbone guided by a mutator that is functionally similar to restriction enzymes. This mechanism makes report generation deterministic and data-centric, while the resulting single-file SPA eliminates the need for a persistent web server.

**Figure 1:**
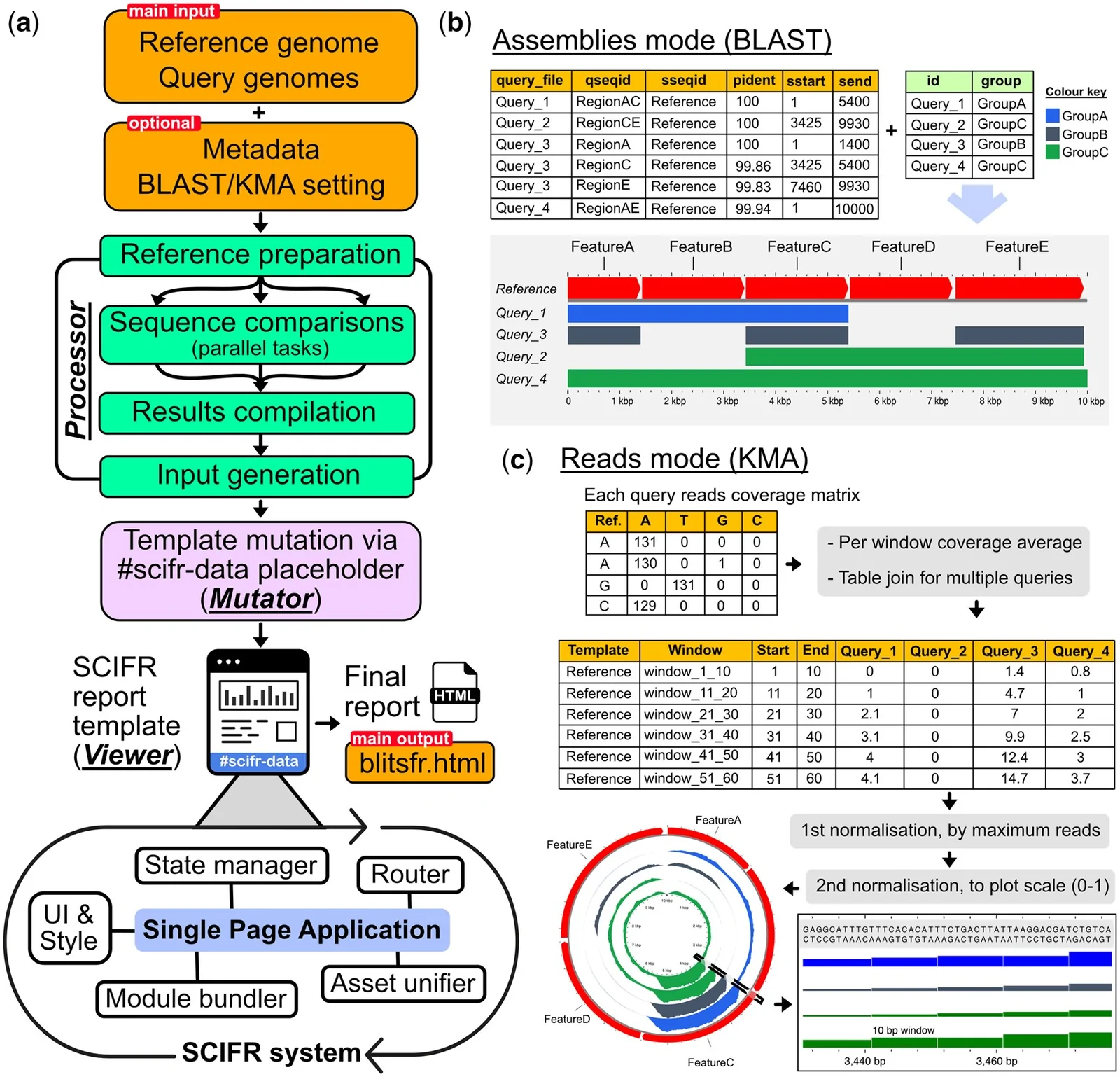
Overview of BLITSFR components and workflow. (a) The BLITSFR pipeline was developed using SCIFR system, which comprises three main components: *Processor, Mutator, and Viewer.* (b) BLITSFR assemblies mode utilises BLAST (Camacho *et al.* 2009) for sequence similarity searches to generate reference-based hit coordinates, which are rendered as linear or circular genome maps using CGView.js (Grant and Stothard 2026) (c) BLITSFR reads mode uses KMA (Clausen *et al.* 2018) to map query genome reads to a reference, resulting in normalised and scaled read coverage for window-based plotting in CGView.js (Grant and Stothard 2026). The resulting interactive tracks can be coloured and sorted based on the provided metadata.

To facilitate immediate deployment and extensibility for SCIFR, a React-based boilerplate was developed and made available via Node.js *create-scifr* package. The boilerplate incorporates essential JavaScript libraries for asset builder and unifier, user interface, and application state management. It supplies a ready-to-run development server, interactive demonstration pages, and a minimal state-management skeleton, enabling developers to scaffold the report template immediately. SCIFR is available as an open-access resource on npm (https://www.npmjs.com/package/create-scifr) and GitHub (https://github.com/nalarbp/scifr).

### 2.2 Development of BLITSFR visualisation template

The BLITSFR visualisation template was developed using SCIFR, initiated by invoking the *npx create-scifr blitsfr* command following Node.js (https://nodejs.org) installation to scaffold the project, pull in dependencies, and create the initial folder structure. Development then proceeded by launching the SPA development server with *npm run dev*, which enabled real-time editing and customisation of the React components through hot module reloading. Because BLITSFR is visualisation-centric, the input template requirements were designed to align with the CGView.js (Grant and Stothard 2026) visualisation library, so the input data structure includes a CGView object and query metadata for user-defined sorting and colouring. The TanStack.js library (https://tanstack.com/table/latest) was used to display the metadata table, linked to the CGView.js APIs using Jotai.js (https://jotai.org/) to create a reactive control system that synchronises the table and the CGView object. Finally, *npm run build* was performed to compile the SPA into a single, self-contained HTML file serving as the SCIFR template, with the resulting input schema handed to the processor developer.

### 2.3 Development of BLITSFR data processor

BLITSFR utilises Nextflow to streamline sequence comparisons and generate input data for the report template. The process begins with indexing the reference genome in GenBank format using *makeblastdb* or *kma_index*, followed by parallel alignment of query sequences. In assemblies mode, nucleotide similarity searches are performed using BLASTn (Camacho *et al.* 2009), resulting in a similarity hit table and alignment coordinates relative to the reference (Fig. 1b). Conversely, read mapping mode uses the fast kmer-based KMA tool (Clausen *et al.* 2018) to calculate query coverage at each reference base, which is then averaged and aggregated into user-defined windows for visualisation as a histogram plot (Fig. 1c). The resulting alignment hits are tabulated and processed to produce map objects for rendering in CGView.js (Grant and Stothard 2026).

When query metadata is provided, it is embedded into the input data for persistent inclusion within the template, though users may also load metadata during the browser session. The Nextflow workflow preserves intermediate outputs, including BLAST reference databases, KMA indices, raw and filtered hit tables, and CGView map JSON files compatible with Proksee (Grant *et al.* 2023), to support reuse and downstream analysis. The final input data payload is then injected into the built template.

### 2.4 Benchmarking SCIFR

To assess the broader application of SCIFR, we compared it with existing tools by developing functionally identical interactive reports using three HTML generation approaches: SCIFR (React-based), Jinja2 v3.1.6 (Python templating), and Quarto v1.7.31 (R-Markdown templating). Each report implemented identical interactive features, including a sortable data table using DataTables.js v1.13.4 and interactive charts using Chart.js v4.2.1, with all JavaScript libraries loaded locally to ensure consistency across tools. Reports were generated using a COVID-19 dataset [25] containing 485 K original data points, systematically scaled to different sizes: 500, 1 K, 2 K, 4 K, 8 K, 16 K, 32 K, 64 K, 128 K, 256 K, and 485 K. Report generation time and output file size were measured and averaged across five iterations to account for system variability. All benchmarks were conducted on a MacBook Pro M3 with 16GB RAM under controlled conditions, with system caches cleared and a delay applied between measurements sessions to ensure consistent baseline performance.

### 2.5 Code development and data availability

The tools, source code and test datasets for BLITSFR and SCIFR are open access and accessible at https://github.com/nalarbp/blitsfr and https://www.npmjs.com/package/create-scifr. The template examples and other tools developed using SCIFR are showcased in https://scifr.fordelab.com.

Upon the revision and maintenance of BLITSFR and SCIFR, large language models have been used to improve the quality of the source code. The authors reviewed, validated and are fully responsible for the accuracy of the final code published.

## 3 Results and discussions

### 3.1 BLITSFR tool overview

BLITSFR is a Nextflow-based command-line tool designed to explore genomic region variation by comparing and visualising multiple query sequences against a reference genome. The tool accepts a GenBank reference file along with one or more query FASTA or FASTQ files and performs sequence alignments using BLAST (Camacho *et al.* 2009) for assembled genomes or KMA (Clausen *et al.* 2018) for read mapping (Fig. 1a). Leveraging Nextflow, BLITSFR distributes processing tasks such as database indexing, sequence alignments, and report generation for efficient comparison of a large number of genomes. The final results are visualised as interactive genome comparison tracks rendered using CGView.js (Grant and Stothard 2026) displaying genome reference features, query tracks and associated metadata within a self‑contained HTML report generated using SCIFR. The report allows users to rearrange tracks, colour them using metadata columns and highlight genomic regions of difference and conservation. Users can generate high-resolution circular and linear comparison images suitable for publication while processing data entirely on their local machine for convenience and privacy. Furthermore, the self-contained format facilitates easy distribution via email, cloud storage, or embedding in web interfaces for better portability.

### 3.2 Use case example 1: revealing the conservation of the *vanB* operon carried by Tn1549-like transposon among Queensland VREfm ST78

To demonstrate the functionality of BLITSFR, we performed comparative genomics on 184 *Enterococcus faecium* ST78 genomes, comprising 58 strains from a previous study (Permana *et al.* 2023) and further 126 *E. faecium* ST78 retrieved from the AllTheBacteria collection (Hunt *et al.* 2026). We compared these genomes against a complete *vanB* ST78 reference genome (Accession NZ_CM151288.1) using BLITSFR assembly mode. The resulting interactive report enabled rapid visual comparison, sorting, and colour-coding of the *E. faecium* genomes according to the provided metadata, facilitating swift identification of both conserved and variable regions across the collection (Fig. 2). For demonstration purpose, we sorted and coloured the genomes by country (Fig. 2a) and *van* type (Fig. 2b). Several regions of difference (RODs) were observed, with these regions largely conserved in Queensland strains but missing in strains from other countries. One distinct ROD was identified as a Tn1549-like transposon carrying the *vanB* operon, which distinguishes the Queensland ST78 strains from those in other countries. Comparison with the canonical Tn1549 reference using Clinker (Gilchrist and Chooi 2021) revealed that the Queensland transposon contains an IS110 element inserted between *vanW* and *vanH*. These comparisons completed in under two minutes on our test laptop (MacBook Pro M3) and in less than one minute on a high-performance computing cluster using 24 CPUs, demonstrating the usability of BLITSFR for rapid exploration and iteration using local resources.

**Figure 2:**
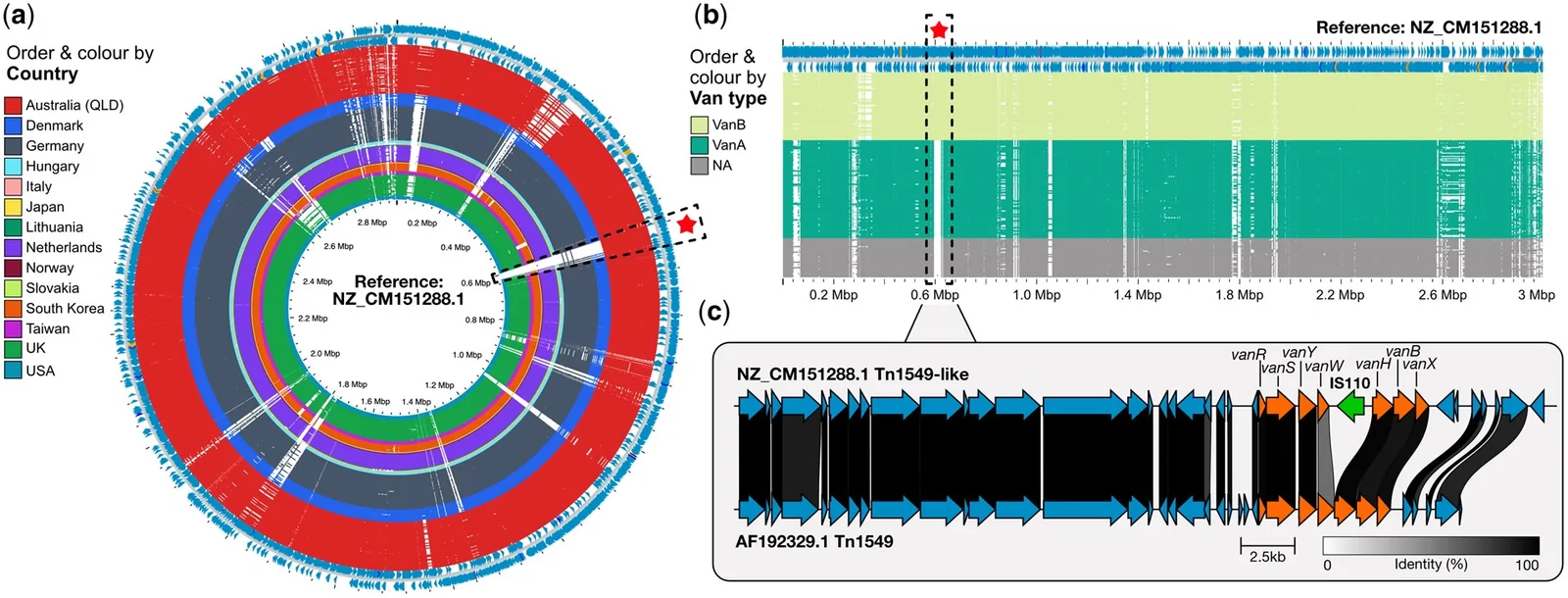
Comparative genome mapping of 184 *Enterococcus faecium* ST78 genomes using BLITSFR assembly mode. A circular genome map of *E. faecium* reference NZ_CM151288.1 displays BLAST hits from 184 query genomes sorted and coloured by country (a) and Van type (b). Several genomic regions of difference were revealed with a distinct Tn1549-like region highlighted. (c) Comparison with canonical Tn1549 transposon sequence using Clinker (Gilchrist and Chooi 2021) showed IS110 (green arrow) present between *vanW* and *vanH* genes within the *vanB* operon (orange arrow).

### 3.3 Use case example 2: visualising the enriched read coverage of the tandem array Tn3505 in induced resistant *Corynebacterium diphtheriae*

Beyond the presence/absence maps generated in assemblies mode, BLITSFR’s reads mode enables investigation of structural variation through read depth plotting, which can resolve copy-number changes and sequence boundaries that presence/absence data alone cannot capture. To demonstrate this feature, we compared sequencing reads from induced and uninduced β-lactam-resistant *Corynebacterium diphtheriae* strains against the CP029644 reference genome, which carries a tandem array of three repeated *prpABC*-*IS*3505 clusters known to expand in copy number under induced conditions (i.e. exposure to meropenem) (Forde *et al.* 2021).

Mapping average read depth across 200 bp windows revealed a marked, localised increase in coverage over the *prpABC* cluster in induced strains relative to uninduced controls (Fig. 3a), consistent with meropenem-driven tandem amplification of Tn3505. Tn3505 carries *prpC*, which encodes the low-affinity penicillin-binding protein Pbp2C in strain BQ11; amplifying this gene is proposed to boost enzyme production enough to overcome meropenem’s inhibitory action, explaining the strain’s shift from sensitive to high-level carbapenem resistance.

**Figure 3:**
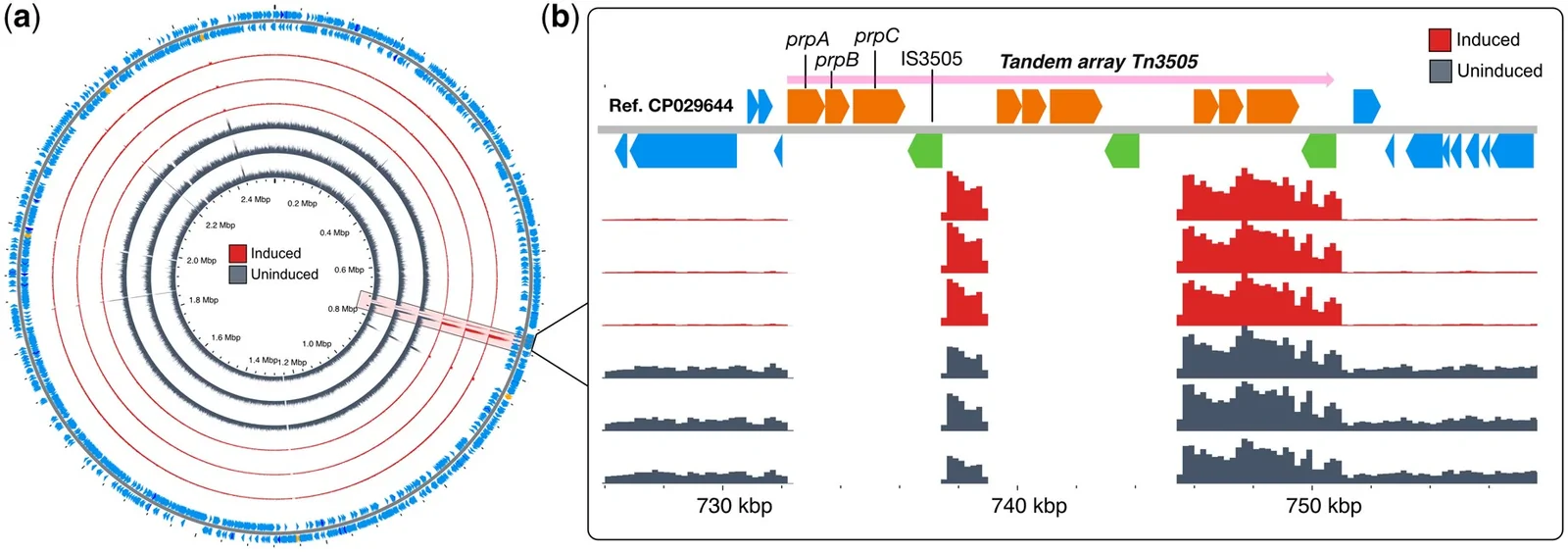
Comparative genome mapping and read coverage analysis of β-lactam resistant *Corynebacterium diphtheriae* using BLITSFR reads mode. (a) A circular genome map of *C. diphtheriae* reference CP029644 with rings representing read coverage for induced (red) and uninduced (dark gray) strains across three replicates. (b) A linear zoom of a genomic region highlighting the Tn3505 tandem array, comprising of three repeated clusters of *prpA*, *prpB*, *prpC*, and IS3505. The induced strains exhibit significant elevation in read coverage, averaged per 200 bp windows across all replicates compared to the uninduced condition relative to the baseline. The pattern suggests an increase in copy number or abundance of this mobile genetic element associated to the resistance.

As with assemblies mode, this analysis was completed in under three minutes on the test machine, underscoring BLITSFR’s speed for rapid, exploratory investigation of structural variants.

### 3.4 Processing scalability and browser-responsiveness

To evaluate BLISTFR, we assessed performance across multiple dataset sizes using subsets of 10–1000 *E. faecium* genomes retrieved from the AllTheBacteria collection (Hunt *et al.* 2026) (Fig. 4a–d, Supplementary Table S1, available at supplementary material  *Bioinformatics Advances* online). Evaluation metrics derived from Nextflow trace reports focused on total duration (Fig. 4a) and maximum peak RAM usage (Fig. 4b), with each measurement averaged over three repetitions. The interactive reports (*Viewer*) were assessed for file size (Fig. 4c) and initial loading time to interactivity (Fig. 4d), defined as the time from initial map loading to the point at which the report is ready for user interaction. Additionally, several interactive functions such as colour mapping and sorting, were evaluated via browser developer window following the initial render.

**Figure 4:**
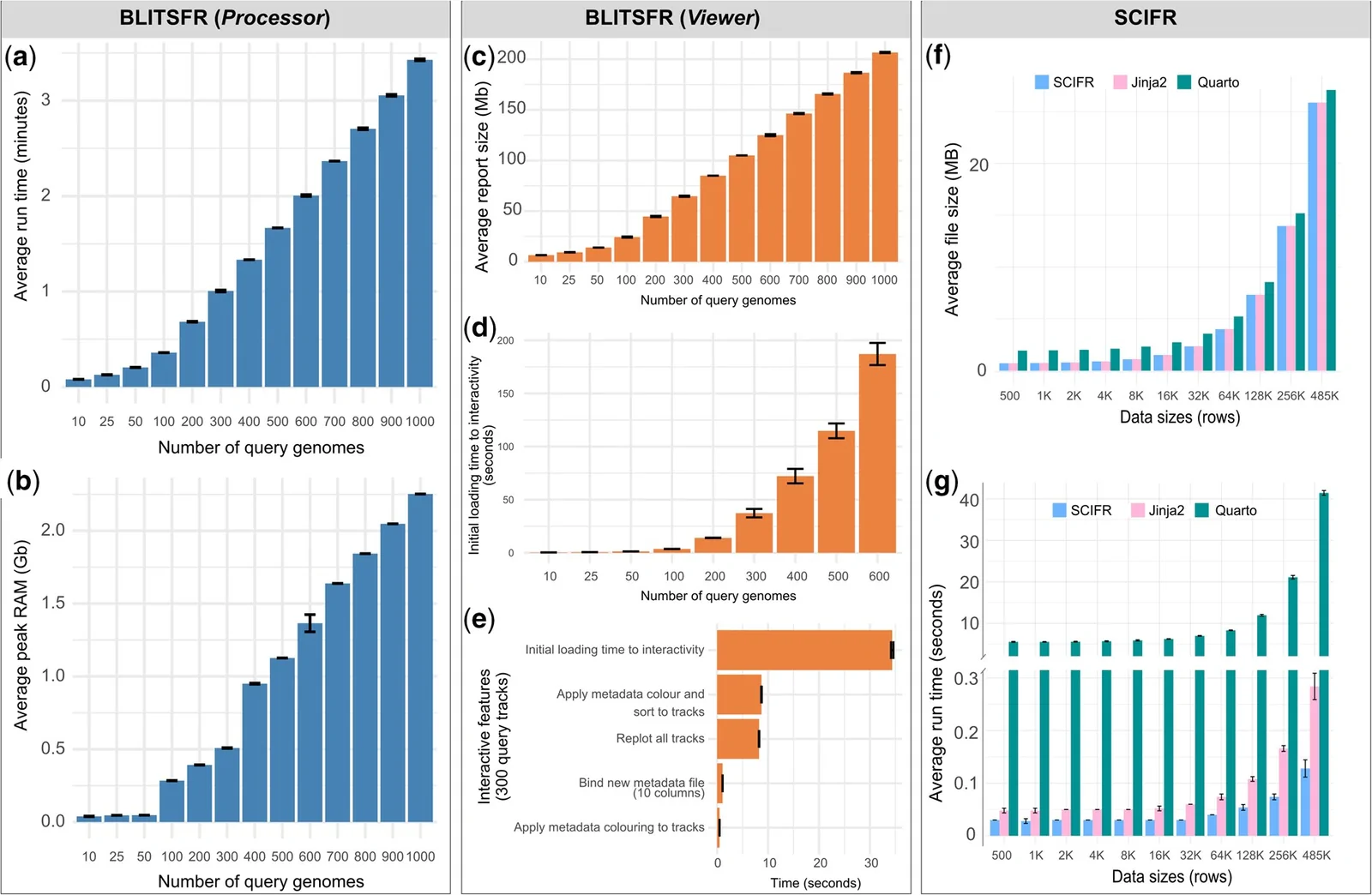
Performance of BLITSFR across different numbers of query genomes (10–1000 *E. faecium* genomes). BLITSFR processor runtime (a) and peak memory (b) scale linearly with the number of query genome, with 1000 genomes requiring under 4 minutes of runtime and approximately 2.5 GB of RAM. BLITSFR report (Viewer) likewise scales linearly in file size (c) and initial loading time to interactivity (d). The initial map creation includes whole input data parsing, and the browser became non-responsive beyond 600 tracks, and data were therefore not collected past this point. However, compared to the initial loading, subsequent interactive action such as track colouring, sorting and metadata binding is significantly faster (e). Comparison of SCIFR, Jinja2, and Quarto across a range of dataset sizes (500–485 k data points) showing average output-file size (f) and report-generation time for each tool across the dataset sizes (g).

The results demonstrate that all computational metrics increase linearly with the number of query genomes. Processing time scales effectively, with 1000 genomes completed in under 3 minutes and peak RAM averaging 2GB. However, the bottleneck resides in the viewer, which is constrained by the rendering load required to visualise the genomic features: browser responsiveness degrades progressively as track and feature counts increase, and the report becomes non-responsive beyond 600 genomes, beyond which further data could not be collected (Fig. 4d). The initial render represents the longest process due to data parsing and map generation, while post-initiation interactivity such as colouring, sorting, and rebinding metadata is significantly faster (Fig. 4e, Supplementary table S2, available at supplementary material  *Bioinformatics Advances* online), demonstrating the benefit of interactive features for data exploration and visualisation. Overall, interactive use remains under 1 minute for visualising genomes with fewer than 300 tracks, though this performance is dependent on the number of features included in the plot. Shorter references or sparse comparison results, which produce fewer rings or features, allow practical interactive use without triggering browser freezing.

### 3.5 Feature comparison with existing tools

BLITSFR overcomes several limitations of existing comparative genomic visualisation tools such as BRIG (Alikhan *et al.* 2011), CCT (Stothard *et al.* 2019), CGView Server (Grant and Stothard 2008), and Proksee (Grant *et al.* 2023). While all these tools are built on the CGView family of viewers, each implements it differently. BRIG wraps the Java CGView renderer in a desktop GUI, and CCT drives the same renderer from the command line; both generate static images and changing the view requires re-running the rendering pipeline. CGView Server, now superseded by Proksee, uses the interactive CGView.js library but operates as a centralised web service that requires data upload and processing on a remote server with limited storage space for users.

BLITSFR provides a unified, end-to-end read-based coverage analysis and visualisation that distinguishes it from existing tools. Unlike BRIG and Proksee, which requires users to provide pre-generated SAM/BAM files and plot data from other tools to create read coverage visualisation, BLITSFR integrates database indexing, read coverage tabulation, user-defined window normalisation, and CGView.js visualisation into a streamlined process. This utility enables users to seamlessly investigate read depth distribution along the reference region across multiple queries, a utility that is useful for resolving copy number variations and mobile genetic element boundary structures that BLAST similarity searches alone often fail to capture.

BLITSFR uniquely combines command-line automation with self-contained client-side interactivity, enabling portability and easier integration with other pipelines, while maintaining the ability to sort, filter, and rearrange tracks by metadata without re-analysis. In contrast to web server-based tools, BLITSFR runs locally, allowing users to leverage their own computational and storage resources without transferring any data to external parties, thus enabling scalable analysis and open tool distribution. Furthermore, BLITSFR preserves intermediate results from multiple processing stages, supporting data governance for comprehensive analysis. Table 1 summarises the key features of BLITSFR compared to BRIG, CCT, CGView Server, and Proksee.

**Table 1:** Comparison of the BLITSFR features to BRIG, CCT, CGView Server, and Proksee.

Feature^a^	BLITSFR (This study)	BRIG (Alikhan *et al.* 2011)	CCT (Grant *et al.* 2012)	CGView Server (Grant and Stothard 2008)	Proksee (Grant *et al.* 2023)
Rendering engine	CGView.js	CGView (Java)	CGView (Java)	CGView (Java)	CGView.js
Interface	Command line + interactive report	Desktop GUI + command line	Command line	Web service	Web service
Main implementation language	Nextflow + JavaScript	Java	Perl + Java	Java	Ruby + JavaScript
Execution location	Local (client-side viewer)	Local desktop	Local (or Docker)	Remote server	Remote server
Requires data upload to third party	No	No	No	Yes	Yes
Built-in sequence comparisons	Yes (BLAST)	Yes (BLAST)	Yes (BLAST)	Yes (BLAST)	Yes (BLAST and other tools on server)
Built-in read mapping	Yes (KMA)	No (but accept SAM file from external tool)	No	No	No
Output format	Single self-contained interactive HTML file	Static images (PNG, JPG, SVG)	Static images (PNG, JPG, SVG)	Static images (PNG, JPG, SVG)	Static images and map JSON via export
Output interactive	Yes (offline)	No	No	No	Yes (online)
Post-generation track reordering, recolouring, and filtering	Yes (locally, in viewer)	No (re-render required)	No (re-render required)	No (re-render required)	Yes (online)
Metadata-driven track colouring/sorting	Yes	No	No	No	Not stated
Third party pipeline integration	Yes	Limited (CLI in fork)	Yes (scriptable)	No	Not stated

a Feature comparison based on published descriptions and public documentation as of 15 July 2026. Software versions were not fixed; features may have changed since.

### 3.6 Comparison of SCIFR system with existing templating workflows

The complex interactive state management in BLITSFR is enabled by SCIFR, a design pattern we argue is extensible for the development of other bioinformatics tools. To assess this, we compared SCIFR against two established HTML report generation workflows: Jinja2 and Quarto (R-markdown version). We generated interactive reports featuring identical charts and tables visualising COVID-19 surveillance data (Mathieu *et al.* 2020) across varying dataset sizes and for each workflow and dataset, measured the output file size (Fig. 4e) and the report generation time (Fig. 4f).

All three workflows generated files of comparable size, which scaled linearly with the volume of input data (Fig. 4e), indicating that data embedding is the primary determinant of final report file size. However, SCIFR demonstrated slightly faster report generation than Jinja2 and Quarto across all dataset sizes (Fig. 4f), an efficiency that stems from SCIFR’s architecture separating template compilation from data injection. As a result, report generation is streamlined to simply inserting data into pre-built templates, reducing overall creation time. Additionally, because SCIFR is a JavaScript-based workflow, it avoids the need to convert data structures from languages such as R (used in Quarto) or Python (used in Jinja2) into JavaScript for browser compatibility.

### 3.7 Limitation and future directions

Nextflow enables BLITSFR to perform large-scale sequence comparisons efficiently, scaling to large number of genomes. However, the current HTML report visualises each genome as an individual track, which is technically limited to a maximum of a few hundred tracks for meaningful visualisation and interpretation. Since all visualisation logic runs client-side, larger or complex datasets can exceed browser memory limits, causing slowdowns or browser freezes. Additionally, report size scales proportionally with the amount of input data (Fig. 4c and f). To mitigate these issues, future developments will incorporate a pre-clustering step to visualise only the most representative tracks while retaining information from the original query list, such as by aggregating redundant records and implementing data pagination to load subsets on demand.

The SCIFR system demonstrates its capacity to support the development of other bioinformatics tools, marking a significant advancement in interactive reporting by unifying speed, scalability, and usability within a single-file SPA format. Ongoing development is directed toward enabling the import and export of input payloads via browser-based indexed databases to facilitate workspace persistence and resumption. As bioinformatics datasets continue to grow in complexity, SCIFR offers a flexible foundation for next-generation interactive reporting, supporting enhanced data interpretation and promising broader compatibility and collaboration.

## Supplementary Material

vbag282_Supplementary_Data

## Data Availability

The tools, source code and test datasets for BLITSFR and SCIFR are open access and accessible at https://github.com/nalarbp/blitsfr, https://github.com/nalarbp/scifr and https://www.npmjs.com/package/create-scifr.
